# Screening mild cognitive impairment using aspects of personal, social, and functional lifestyle: Machine Learning Approaches

**DOI:** 10.1371/journal.pone.0334704

**Published:** 2025-10-24

**Authors:** Kyle Masato Ishikawa, Matthew Uechi, Hyeong Jun Ahn, Eunjung Lim

**Affiliations:** 1 Department of Quantitative Health Sciences, John A. Burns School of Medicine, University of Hawaii at Manoa, Honolulu, Hawaii, United States of America,; 2 Department of Geriatric Medicine, John A. Burns School of Medicine, University of Hawaii at Manoa, Honolulu, Hawaii, United States of America; University of Thessaly Faculty of Medicine: Panepistemio Thessalias Tmema Iatrikes, GREECE

## Abstract

**Objective:**

Mild cognitive impairment (MCI) signals cognitive decline beyond normal aging and increases dementia risk. Early identification enables preventative interventions, yet many patients in primary care go undetected. This study examines whether machine learning (ML) models can predict MCI using routinely collected personal, social, and functional lifestyle factors and identifies the most important predictors.

**Methods:**

Data from round 2 and 3 of the National Social Life, Health, and Aging Project was used, including 4,586 older adults with complete Montreal Cognitive Assessment (MoCA) scores. Predictors included demographics, childhood experiences, health behaviors, psychosocial measures, and functional difficulties. Eight ML models—including elastic net, multivariate adaptive regression splines, random forest, oblique random forest, boosted trees, decision trees, and a stacked ensemble—were trained and tuned using repeated cross-validation, with 20% of the dataset withheld for final testing. Model performance was assessed using area under the receiver operator curve (AUROC), accuracy, sensitivity, specificity, and Matthew’s correlation coefficient (MCC).

**Results:**

Most models achieved good discrimination (AUROC > 0.8), with the stacked ensemble performing best (AUROC = 0.823; MCC = 0.462). The best individual model was logistic regression (AUROC = 0.818). Across models, key predictors of MCI included age, ethnicity, functional difficulties, social disconnectedness, and perceived stress.

**Discussion:**

Logistic regression outperformed more complex machine learning models, providing the best combination of predictive accuracy and interpretability for identifying MCI. Across models, age, ethnicity, functional difficulties, social disconnectedness, and stress consistently emerged as key predictors, highlighting their central role in cognitive health. These findings suggest that psychosocial and functional measures can serve as practical indicators for those who need to be screened early for MCI, offering an opportunity for timely intervention and support. However, future work should include longitudinal data and clinical diagnoses to validate and refine these predictive tools.

## Introduction

Mild cognitive impairment (MCI) marks a steeper decline in cognition than one would expect from normal aging. While MCI serves as a precursor to dementia, individuals with this condition are still able to manage their daily activities. It is estimated that 15–20 percent of adults over the age of 60 experience MCI, with eight to 15 percent progressing to dementia annually [1]. This underscores the importance of early detection, as it may allow for preventative lifestyle interventions.

It is estimated that 29% to 76% of patients with dementia may go undetected in primary care, and as of 2020, the U.S. Preventive Services Task Force concluded there is insufficient evidence to assess the benefits and harms of screening for cognitive impairment in older adults [2]. The Centers for Medicare & Medicaid Services recognizes the importance of screening for cognitive impairment and thus includes checking for cognitive impairment as part of the Medicare Annual Wellness Visit, an annual health evaluation for all Medicare beneficiaries focused on providing preventative care [3]. That said, even brief assessments of cognitive function require some time from busy clinicians, and it may be beneficial to identify a subset of older adults who are at highest risk for the development of cognitive impairment.

The Montreal Cognitive Assessment (MoCA) is a validated 30-point screening tool commonly used to assess cognitive decline [4]. Researchers have used the MoCA to identify risk factors associated with MCI or dementia. For example, Kotwal et al found that small network size (close family and friends), high frequency of interaction within that network, low social strain, and low community involvement were linked with a higher risk of MCI [5]. In more detail, women tended to perceive less social support, while men were more likely to engage in social activities with neighbors and family. Another study found that MoCA scores decline with age and differ by gender, education, and race/ethnicity [6], with poor physical health, inability to do daily living activities, and depression being associated with lower scores.

Machine learning (ML) models have previously been used to predict cognitive decline and determine possible risk factors. Using the National Health and Nutrition Examination Survey, Li et al selected 10 features and used various models to predict cognitive impairment [7]. The generalized linear model performed the best with an area under the receiver operator curve (AUROC) of 0.779. A similar study using lifestyle behaviors and blood work measurements achieved an AUROC of 0.810 with an extreme gradient boosting model and identified *instrumental activities of daily living* as the strongest predictor of MCI [8]. Another study developed a ML framework to detect clinically diagnosed MCI or dementia by utilizing demographic data, functional activities, biomarkers, medical imaging, and screening tools such as the MoCA and Mini-Mental State Exam (MMSE) as predictors [9]. Their model achieved an AUROC of 0.970 in identifying MCI. Without the use of neuroimaging features, another study in China achieved an AUROC of 0.9282 using only demographic and lifestyle features, along with four different cognitive screening assessments [10]. This study distinguishes itself by including aspects of loneliness developed by researchers at Cornell University [11], depression, stress, anxiety, and family upbringing to determine risk factors that would warrant further MCI examination. It does not contain any screening assessments directly related to cognition.

Therefore, it is crucial to assess how the risk factors identified in literature perform in a real-world screening context, with the MoCA serving as a proxy for MCI. This exploratory study will utilize ML models to predict cognitive status based on personal, social, and functional lifestyle factors, comparing the effectiveness of each model to logistic regression. In particular novel measurements of objective and subjective loneliness, as created and validated by Cornwell and Waite (11), will be utilized by progressively more complex tree-based models. The findings will provide valuable insights into lesser-known characteristics associated with MCI and help clinicians target individuals for early MCI screening.

## Methods

### Data

The National Social Life, Health, and Aging Project (NSHAP) is a national longitudinal survey that focuses on social, cognitive, and lifestyle changes as participants age [12,13]. This study used rounds two and three of the NSHAP because these rounds included the MoCA. The NSHAP data was downloaded from the Inter-university Consortium for Political and Social Research repository on July 3, 2023. This study was considered *not human subjects research* by the Office of Research Compliance at the University of Hawaii and, therefore, not subject to the institutional review board’s review.

### Measures

The MoCA is a 30-point questionnaire used to screen individuals for MCI [4]. It assesses participants across eight cognitive domains: visuospatial abilities, executive function, naming, short-term memory, attention, language, abstraction, delayed recall, and orientation to time and place. A score below 23 indicates *MCI* [14], while a score of 23 or higher suggests *normal* cognitive function.

Demographics and characteristics related to personal, social, and functional lifestyle were used to predict MCI. Composite variables include the NSHAP Depressive Symptoms Measure (NDSM) based on the Center for Epidemiologic Studies Depression Scale (CES-D) [15], Perceived Stress Scale 4 (PSS4) [16], the Hospital Anxiety and Depression Scale (HADS) [17], functional difficulties, and measures of social disconnectedness (SD) and perceived isolation (PI) [11]. Social disconnectedness describes objective characteristics of the interviewee’s personal connections and community involvement, while PI describes the interviewee’s sentiment toward their social network. Since the NDSM encapsulates depression, only the HADS questions related to anxiety were used. A full list of predictors and the variables used to create them are listed in Table 1. The color of each variable name indicates if it was inputted into models as a continuous or categorical variable.

**Table 1:** Model predictors.

Demographic	• **Age**. • ***Gender***. • ***Ethnicity***. (White, Black, Hispanic, or other) • ***Income***. Total household income. • ***Marital***. • ***Employment***. • **BMI**. • ***Military***. Have you ever served in the US military?
Personal lifestyle	• ***Smoke***. Do you smoke cigarettes, cigars, or a pipe? • ***Drink***. Did you drink alcohol in the last three months? • ***Religion***. • ***Happy***. Was family life happy growing up? • ***Financial*.** Was your family well off from age 6–16? • ***Parent***. Did you live with both parents from age 6–16? • ***Health*.** How was your health from age 6–16? • ***Experience*.** Did you experience a violent event from age 6–16? • ***Witness*.** Did you witness a violent event from age 6–16? • ***Think***. How often do you think about sex? • ***Important***. How important is sex? • ***Agree***. How often do you agree to have sex? • **NDSM.** NSHAP Depressive Symptoms Measure based on the Center for Epidemiologic Studies Depression Scale (CES-D). “During the last week…” ◦ I did not feel like eating. ◦ I felt depressed. ◦ I felt everything was an effort. ◦ sleep was restless. ◦ I was happy. ◦ I felt lonely. ◦ people were unfriendly. ◦ I enjoyed life. ◦ I felt sad. ◦ I felt people disliked me. ◦I could not get going. • **PSS4.** Perceived Stress Scale 4. “In the last month…” ◦ how often have you felt that you were unable to control the important things in your life? ◦ how often have you felt confident about your ability to handle your personal problems? ◦ how often have you felt that things were going your way? ◦ how often have you felt difficulties were piling up so high that you could not overcome them? • **HADS**. Hospital Anxiety and Depression Scale. “During the past week…” ◦ I felt tense or “wound up.” ◦ I got a frightened feeling as if something awful was about to happen. ◦ worrying thoughts went through my mind. ◦ I could sit at ease and feel relaxed. ◦ I got a frightened feeling like butterflies in my stomach. ◦ I felt restless as if I had to be on the move. ◦ I had a sudden feeling of panic.
Social lifestyle	• **SD**. Social disconnectedness. ◦ Social network size (five max) ◦ Number of distinct roles in social network ◦ Average interaction with social network within a year ◦ Proportion of social network that lives at home ◦ Number of friends ◦ Frequency of attending organized groups ◦ Frequency of socializing with friends or family ◦ Frequency of volunteer work • **PI**. Perceived isolation. “How often…” ◦ can you open up to spouse/partner? ◦ can you rely on spouse/partner? ◦ can you open up to family? ◦ can you rely on family? ◦ can you open up to friends? ◦ can you rely on friends? ◦ do you feel lack of companionship? ◦ do you feel left out? ◦ do you feel isolated?
Functional lifestyle	• ***Physical***. How often do you engage in physical activity? • **Functional**. Summation of functional difficulties which include the following. ◦ Preparing meals ◦ Taking medication ◦ Managing money ◦ Shopping for groceries ◦ Performing light housework ◦ Using a telephone ◦ Walking across a room ◦ Walking one block ◦ Dressing ◦ Bathing or showering ◦ Eating ◦ Getting in or out of bed ◦ Using the toilet ◦ Driving a car during day ◦ Driving a car during night

**Note**: Predictors that list subitems are composite variables.

Variables that are *italicized* are categorical variables.

### Study sample

**Fig 1:**
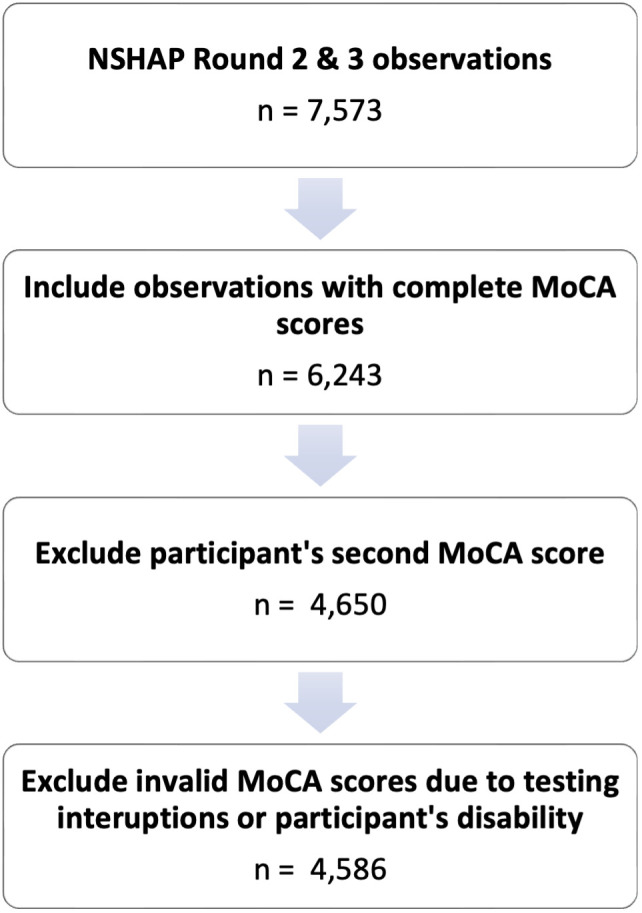
Study flowchart.

The MoCA was included in Rounds 2 and 3 of the NSHAP, and participants were included in this study if they answered all MoCA questions. To minimize any potential learning effect, only the first MoCA score of each participant was used. Scores were excluded if the interviewer noted any disruption during the assessment or if a disability was present that would prevent the participant from completing the assessment fairly. The inclusion and exclusion of study participants are documented in Fig 1.

### Machine learning workflow

#### Data spending.

Eighty percent of the dataset was selected at random and used as the *train set* and the remaining 20% of data was used as the *test set* to evaluate model performance at the end of the workflow. The test set was completely isolated from the training set and meant to simulate “unseen” data. It was not involved with model preprocessing, tuning, calibration, or final training. The training set was further divided into five cross-fold validation sets. This process was repeated twice to eliminate any bias from the initial data splitting, resulting in ten validation sets used for model tuning.

### Data preprocessing

Preprocessing steps included aggregating small categorical predictor levels, imputing missing values, centering and scaling continuous predictors, creating dummy variables for categorical predictors, or creating interaction terms. Parameters for preprocessing steps were calculated using only the training data and then applied to the validation/test sets. For example, when a continuous predictor was centered and scaled, the mean and standard deviation were calculated using only the training data, and these parameters were applied to the test data. The following preprocessing were applied to all models.

- Predictors with a relatively large number of missing responses had those responses recoded as “unknown” in case they provided meaningful information. For example, patients who refused to answer if they witnessed violence in their childhood may have an association with MCI. If responses were missing at random, then recoding these values into an unknown category will not improve performance, but it will enable regression models to use these observations and information from other variables. Predictors that were recoded include household income, drank within three months, military service, perceived importance and frequency of sex, religion, and childhood questions regarding happiness, financial status, living with parents, health, experiencing and witnessing violence. Little’s test was performed on a subset of predictors, including witnessing violence, age, gender, ethnicity, and marital status. The test revealed that these variables were not missing completely at random (p < 0.001). Other predictors, in conjunction with demographics, may share a similar pattern.
- Missing values for all the other predictors were imputed using a bagged trees method.
- For each categorical variable, levels comprising less than five percent of the data were combined into an “other” category to prevent these observations from exerting high leverage on the model. Consequently, if these small groups have characteristics that are associated with MCI, they will not be represented in the results.
- Due to the class imbalance of the outcome variable (with MCI comprising only 27.1% of the data), the synthetic minority over-sampling technique (SMOTE) was used to generate observations for the event class based on the feature space of similar observations [18]. This approach reduced the model’s bias toward predicting normal cognition and allowed the models to account for features from the event class. Observations were created until the event class represented three-fourths of the non-event class which nearly doubled the number of event observations. Synthetic datapoints were limited to reduce the amount of noise introduced to the data. This was particularly useful in cases where the feature space of the minority class overlapped with the majority class.
- Lastly, variables with high correlation were NOT removed so that individual models could include, remove, or regularize them. For example, elastic net improves the logistic regression model by adding penalization parameters which shrink or eliminate poorly performing or correlated predictors. By including all predictors, models such as oblique random forest could utilize interaction terms with distinct relationships.

For this study, we utilized the following ML methods: logistic regression, elastic net, multivariate adaptive regression spline, decision tree, random forest, oblique random forest, boosted tree, and stacked ensemble. The elastic net model used a regularization technique that required continuous predictors to be normalized, centered to a mean of zero, and scaled to a standard deviation of one. Both the elastic net and boosted trees model required a numeric input matrix, so all categorical variables were transformed into dummy variables that were represented by ones and zeros.

### Model tuning

The best set of hyperparameters for each model were selected based on their average performance within the validation sets. The performance metric used to select the best set of hyperparameters was the AUROC. During grid search, ANOVA was used to eliminate sub-optimal hyperparameters and reduce the computational load required for tuning [19].

For the stacked ensemble, the probability predictions of all tuned models were inputted into the ensemble’s *super learner* which was a LASSO regression model. Cross-validation from training data was used to determine the optimal penalty parameter which, in turn, determined whose model predictions would be included in the ensemble and how much influence they had on the final prediction.

### Optimal probability threshold

**Fig 2:**
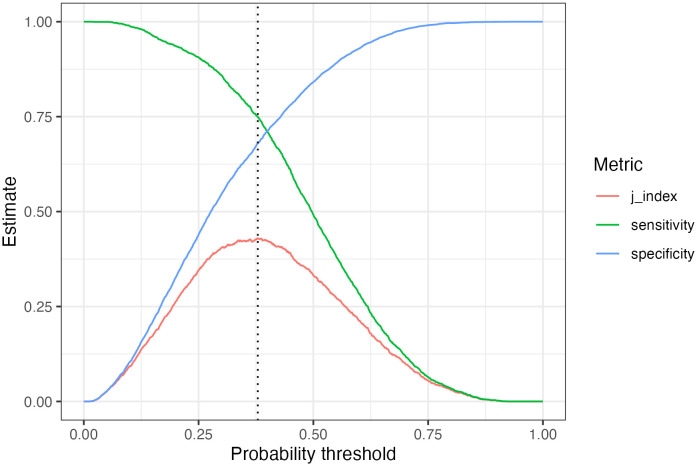
Random forest optimal probability threshold based on validation predictions.

By default, all models use a probability threshold of 0.5 to classify normal or MCI status. This study defines the optimal threshold as the probability that maximizes the *j-index *sensitivity+specificity−1, resulting in a score that ranges from zero to one. For example, the sensitivity, specificity, and j-index of a random forest model is plotted in Fig 2. These values were determined by the tuned model’s performance on the validation sets. The dashed line indicates the probability threshold where the j-index is maximized. This threshold will be applied to the test set.

### Final evaluation

Performance metrics of AUROC, accuracy, sensitivity, specificity, and Matthew’s correlation coefficient (MCC) were calculated for each model’s predictions on the test set. The MCC ranges from negative one to one, taking into account the balance of both sensitivity and specificity [20]. A MCC of one represents complete agreement between the predictions and truth values and a value of negative one represents complete disagreement.

### Interpretation

Model-specific results were presented for each model. Odds ratios (ORs) were presented for the logistic regression and elastic net models. Since the elastic net required continuous predictors to be normalized, centered, and scaled, the interpretation of ORs from continuous variables roughly translates to, “the odds of MCI increase or decrease by __% per one standard deviation increase.” For the decision tree model, a logical map was plotted. For the multivariate adaptive regression splines (MARS) model, the regression equation that includes hinge functions was presented. Lastly, variable importance plots (VIPs) using the permutation method were presented for the random forest, oblique random forest, and boosted trees models. This method randomly permutated the values of each predictor and recorded the drop in performance. If a predictor did not contain useful information, then permutating its values was not expected to lower performance.

### Statistical analysis and software

Descriptive analysis was performed on the training data to gain an intuition on the differences between participants with normal and mildly impaired cognition. Pearson’s Chi-squared tests were used to compare categorical variables and Wilcoxon rank sum tests were used to compare continuous variables.

Data wrangling, manipulation, and plotting were performed using packages from the *Tidyverse* ecosystem [21]. The ML workflow was facilitated by packages from the *Tidymodels* ecosystem [22]. Tables which included statistics were created using the *gtsummary* package [23]. The SMOTE was performed by the *themis* package [24] and probability calibration was calculated using the *probably* package [25]. All code was implemented in R version 4.4.1 and reproducibility was managed by the *targets* package [26].

## Results

The study data included 4,586 individuals from round 2 and 3 of the NSHAP who had complete MoCA scores. Table 2 compares the characteristics of cognitively normal and impaired adults, encompassing all of the study data. With the exceptions of BMI, smoking status, and experiencing or witnessing violence in childhood, all other characteristics show significant differences between the cognitive groups, indicating that they are strong predictors in the multivariable models.

**Table 2:** Comparison of normal and cognitively impaired adults in study population.

Predictor	Overall N = 4,586^1^	Normal N = 3,343^1^	MCI N = 1,243^1^	p-value^2^
**Gender**				**<0.001**
* male*	2,217 (48%)	1,551 (46%)	666 (54%)	
* female*	2,369 (52%)	1,792 (54%)	577 (46%)	
**Age**	66 (59, 74)	65 (58, 71)	71 (63, 79)	**<0.001**
**Race/ethnicity**				**<0.001**
* White*	3,316 (73%)	2,672 (80%)	644 (52%)	
* Black*	674 (15%)	341 (10%)	333 (27%)	
* Hispanic, non-Black*	439 (9.6%)	220 (6.6%)	219 (18%)	
* other*	144 (3.1%)	101 (3.0%)	43 (3.5%)	
* Unknown*	13	9	4	
**Household income**				**<0.001**
* 0-24,999*	826 (23%)	461 (17%)	365 (40%)	
* 25,000-49,999*	939 (26%)	670 (24%)	269 (29%)	
* 50,000-99,999*	1,135 (31%)	923 (34%)	212 (23%)	
* 100k or higher*	761 (21%)	690 (25%)	71 (7.7%)	
* Unknown*	925	599	326	
**Marital status**				**<0.001**
* married*	3,047 (66%)	2,338 (70%)	709 (57%)	
* living with a partner*	151 (3.3%)	104 (3.1%)	47 (3.8%)	
* separated*	74 (1.6%)	44 (1.3%)	30 (2.4%)	
* divorced*	518 (11%)	372 (11%)	146 (12%)	
* widowed*	573 (12%)	335 (10%)	238 (19%)	
* never married*	223 (4.9%)	150 (4.5%)	73 (5.9%)	
**Employment**				**<0.001**
* working*	1,760 (38%)	1,443 (43%)	317 (26%)	
* retired*	2,179 (48%)	1,458 (44%)	721 (58%)	
* unemployed*	138 (3.0%)	106 (3.2%)	32 (2.6%)	
* homemaker*	175 (3.8%)	134 (4.0%)	41 (3.3%)	
* disabled*	325 (7.1%)	195 (5.8%)	130 (10%)	
* Unknown*	9	7	2	
**Body mass index** (BMI)	29 (26, 33)	29 (26, 33)	29 (25, 33)	0.132
* Unknown*	390	233	157	
**Physical activity**				**<0.001**
* never*	874 (19%)	537 (16%)	337 (27%)	
* less than 1 time per month*	432 (9.4%)	319 (9.5%)	113 (9.1%)	
* 1 - 3 times per month*	414 (9.0%)	319 (9.5%)	95 (7.6%)	
* 1 - 2 times per week*	748 (16%)	567 (17%)	181 (15%)	
* 3 or 4 times per week*	956 (21%)	766 (23%)	190 (15%)	
* 5 or more times per week*	1,160 (25%)	834 (25%)	326 (26%)	
* Unknown*	2	1	1	
**Smoke cigarettes**	756 (16%)	530 (16%)	226 (18%)	0.059
**Drank within 3 months**	1,276 (45%)	1,060 (50%)	216 (29%)	**<0.001**
* Unknown*	1,737	1,233	504	
**Military service**	824 (23%)	584 (21%)	240 (27%)	**<0.001**
* Unknown*	988	623	365	
**Think about sex**				**<0.001**
* never*	415 (9.4%)	213 (6.6%)	202 (17%)	
* less than once a month*	869 (20%)	613 (19%)	256 (22%)	
* one to a few times a month*	1,097 (25%)	815 (25%)	282 (24%)	
* one to a few times a week*	1,163 (26%)	902 (28%)	261 (22%)	
* every day*	648 (15%)	508 (16%)	140 (12%)	
* several times a day*	204 (4.6%)	158 (4.9%)	46 (3.9%)	
* Unknown*	190	134	56	
**Importance of sex**				**<0.001**
* not at all important*	1,034 (27%)	728 (26%)	306 (31%)	
* somewhat important*	778 (21%)	577 (21%)	201 (21%)	
* moderately important*	980 (26%)	772 (27%)	208 (21%)	
* very important*	767 (20%)	573 (20%)	194 (20%)	
* extremely important*	225 (5.9%)	161 (5.7%)	64 (6.6%)	
* Unknown*	802	532	270	
**Consensual sex**				**<0.001**
* never*	185 (5.6%)	117 (4.7%)	68 (8.2%)	
* rarely*	102 (3.1%)	70 (2.8%)	32 (3.9%)	
* sometimes*	294 (8.9%)	185 (7.4%)	109 (13%)	
* usually*	840 (25%)	707 (28%)	133 (16%)	
* always*	1,348 (41%)	1,035 (41%)	313 (38%)	
* partner hasn’t wanted sex/does not have partner*	552 (17%)	381 (15%)	171 (21%)	
* Unknown*	1,265	848	417	
**Religion**				**<0.001**
* none*	408 (10%)	335 (12%)	73 (7.1%)	
* Catholic*	1,108 (28%)	783 (27%)	325 (31%)	
* other religion*	803 (20%)	605 (21%)	198 (19%)	
* Protestant*	1,610 (41%)	1,173 (41%)	437 (42%)	
* Unknown*	657	447	210	
**Childhood: Family life was happy**				**<0.001**
* I disagree very much*	193 (5.4%)	137 (5.1%)	56 (6.4%)	
* I disagree pretty much*	332 (9.3%)	247 (9.2%)	85 (9.7%)	
* I disagree a little*	375 (11%)	270 (10%)	105 (12%)	
* I agree a little*	346 (9.7%)	266 (9.9%)	80 (9.2%)	
* I agree pretty much*	1,524 (43%)	1,217 (45%)	307 (35%)	
* I agree very much*	785 (22%)	545 (20%)	240 (27%)	
* Unknown*	1,031	661	370	
**Childhood: Family was well off**				**<0.001**
* not well off at all*	502 (14%)	358 (13%)	144 (16%)	
* not so well off*	1,044 (29%)	765 (29%)	279 (32%)	
* about average*	1,541 (43%)	1,228 (46%)	313 (35%)	
* fairly well off*	396 (11%)	278 (10%)	118 (13%)	
* very well off*	80 (2.2%)	51 (1.9%)	29 (3.3%)	
* Unknown*	1,023	663	360	
**Childhood: Lived with parents**	2,990 (84%)	2,287 (85%)	703 (80%)	**<0.001**
* Unknown*	1,028	663	365	
**Childhood: Health**				**<0.001**
* poor*	34 (0.9%)	19 (0.7%)	15 (1.7%)	
* fair*	193 (5.3%)	112 (4.1%)	81 (8.9%)	
* good*	688 (19%)	474 (17%)	214 (24%)	
* very good*	1,120 (31%)	844 (31%)	276 (30%)	
* excellent*	1,605 (44%)	1,284 (47%)	321 (35%)	
* Unknown*	946	610	336	
**Childhood: Experience violence**	435 (12%)	343 (13%)	92 (10%)	0.053
* Unknown*	957	618	339	
**Childhood: Witness violence**	502 (14%)	385 (14%)	117 (13%)	0.367
* Unknown*	955	617	338	
**Social disconnectedness** (SD)	−0.04 (−0.34, 0.31)	−0.09 (−0.37, 0.25)	0.09 (−0.26, 0.42)	**<0.001**
**Perceived isolation** (PI)	−0.10 (−0.47, 0.35)	−0.15 (−0.47, 0.31)	0.00 (−0.41, 0.49)	**<0.001**
* Unknown*	119	80	39	
**Perceived Stress Scale 4** (PSS-4)	6.00 (4.00, 9.00)	6.00 (4.00, 9.00)	8.00 (5.00, 10.00)	**<0.001**
* Unknown*	910	563	347	
**NSHAP Depressive Symptoms Measure** (NDSM)	4.0 (1.0, 8.0)	3.0 (1.0, 7.0)	5.0 (2.0, 10.0)	**<0.001**
* Unknown*	46	20	26	
**Functional difficulties**	1.0 (0.0, 3.0)	0.0 (0.0, 2.0)	2.0 (0.0, 7.0)	**<0.001**
* Unknown*	42	16	26	
**Hospital Anxiety and Depression Scale** (HADS)	4.0 (2.0, 7.0)	4.0 (2.0, 7.0)	5.0 (3.0, 8.0)	**<0.001**
* Unknown*	1,017	630	387	

^1^n (%); Median (Q1, Q3).

^2^Pearson’s Chi-squared test; Wilcoxon rank sum test.

**Fig 3:**
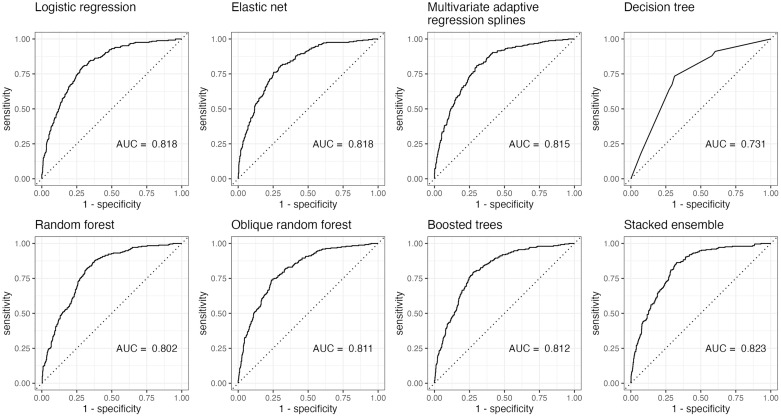
Area under the receiver operator curve for all models.

The finalized hyperparameters of all models are listed in Table 3 and the performance of all models are in Table 4. The receiver operating characteristic (ROC) curve of each model is presented in Fig 3. The stacked ensemble had the had the highest AUROC, accuracy, specificity, and MCC. All models except for decision tree performed moderately well with AUROCs greater than 0.8 on the test set. This result is comparable to the models of Li, Zeng (7) and Ren, Zheng (8) which did not use neuroimaging or cognitive screening assessments as predictors.

**Table 3:** Model packages and final hyperparameters.

Model	R Package	Final Hyperparameters
Logistic regression	stats	NA
Elastic net	glmnet	Penalty = 0.0019 Mixture = 0.623
Multivariate Adaptive Regression Splines	earth	Degree of interaction terms = 1 Prune method = “backward”
Decision tree	rpart	NA
Random forest	ranger	Number of random predictors selected for each node = 4 Trees in the forest = 1,275 Minimum number of observations in a node = 35
Oblique random forest	aorsf	Number of random predictors selected for each node = 25 Trees in the forest = 914 Minimum number of observations in a node = 33
Boosted trees	xgboost	Number of random predictors selected for each node = 5 Trees in the forest = 1,250 Minimum number of observations in a node = 17 Tree depth = 10
Stacked ensemble	stacks	Penalty = 10^−6^

**Table 4:** Model performance.

Model	AUROC*	Accuracy	Sensitivity	Specificity	MCC*
Logistic regression	0.818 (0.788, 0.847)	0.709 (0.681, 0.739)	0.827 (0.780, 0.873)	0.666 (0.631, 0.700)	0.438 (0.384, 0.492)
Elastic net	0.818 (0.790, 0.846)	0.720 (0.692, 0.749)	0.810 (0.760, 0.859)	0.687 (0.654, 0.722)	0.444 (0.387, 0.501)
Multivariate adaptive regression splines	0.815 (0.785, 0.844)	0.707 (0.676, 0.734)	0.819 (0.676, 0.734)	0.666 (0.628, 0.700)	0.431 (0.374, 0.485)
Decision tree	0.731 (0.697, 0.764)	0.700 (0.671, 0.730)	0.734 (0.675, 0.789)	0.688 (0.655, 0.724)	0.379 (0.319, 0.441)
Random forest	0.802 (0.772, 0.831)	0.720 (0.692, 0.748)	0.810 (0.759, 0.857)	0.687 (0.654, 0.721)	0.444 (0.389, 0.500)
Oblique random forest	0.811 (0.782, 0.839)	0.714 (0.685, 0.744)	0.782 (0.731, 0.833)	0.688 (0.653, 0.724)	0.421 (0.362, 0.481)
Boosted trees	0.812 (0.783, 0.841)	0.682 (0.653, 0.712)	0.855 (0.808, 0.897)	0.618 (0.582, 0.654)	0.420 (0.367, 0.473)
Stacked ensemble	0.823 (0.793, 0.849)	0.740 (0.711, 0.766)	0.794 (0.750, 0.849)	0.719 (0.684, 0.750)	0.462 (0.407, 0.518)

*AUROC = Area Under the Receiver Operator Curve; MCC = Matthews Correlation Coefficient.

Ninety-five percent confidence intervals were generated from the 2.5th and 97.5th percentiles of 1,000 bootstrap samples of the test data.

The OR of each predictor from the logistic regression and elastic net models are listed in Table 5. Post-modeling diagnostics determined that the following childhood variables had high correlation (variable inflation factor > 10) – experienced violence, witnessed violence, was healthy, lived with both parents, and was happy. Experienced and witnessed violence in childhood had the strongest Kendall rank correlation (0.49) and both those variables had a negative association with happiness (−0.25 and −0.23, respectively). Categorical predictors were transformed into *dummy encodings* before being modelled in the elastic net, meaning that categorical levels which were “removed” by the elastic net model were actually combined with the reference level (indicated by the dash, -).

**Table 5:** Odds ratios of logistic regression and elastic net models.

Predictor	Logistic OR (95% CI)^1^	Logistic p-value	Elastic OR^1^
**Female**	0.54 (0.45, 0.65)	**<0.001**	0.58
**Age**	1.06 (1.05, 1.07)	**<0.001**	1.68
**Race/ethnicity**			
* White*	–	–	–
* Black*	4.49 (3.64, 5.55)	**<0.001**	4.39
* Hispanic non-Black*	3.96 (3.06, 5.13)	**<0.001**	4.00
* other*	2.32 (1.56, 3.46)	**<0.001**	2.20
**Household income**			
* 0-24,999*	–	–	–
* 25,000-49,999*	0.73 (0.59, 0.92)	**0.006**	0.79
* 50,000-99,999*	0.54 (0.43, 0.69)	**<0.001**	0.60
* 100k or higher*	0.36 (0.27, 0.49)	**<0.001**	0.42
* unknown*	0.88 (0.71, 1.11)	0.282	0.96
**Marital status**			
* married*	–	–	–
* divorced*	1.16 (0.89, 1.51)	0.274	1.08
* widowed*	1.53 (1.18, 1.98)	**0.001**	1.45
* other*	1.13 (0.87, 1.47)	0.345	1.06
**Employment**			
* working*	–	–	–
* retired*	0.95 (0.79, 1.15)	0.614	0.98
* disabled*	1.02 (0.74, 1.41)	0.907	Removed^2^
* other*	0.54 (0.38, 0.75)	**<0.001**	0.58
**Body mass index** (BMI)	1.00 (0.99, 1.01)	0.747	0.99
**Physical activity**			
* never*	–	–	–
* less than 1 time per month*	0.92 (0.69, 1.21)	0.540	0.95
* 1 - 3 times per month*	0.79 (0.58, 1.05)	0.109	0.86
* 1 - 2 times per week*	1.01 (0.80, 1.29)	0.912	1.05
* 3 or 4 times per week*	0.86 (0.68, 1.09)	0.223	0.97
* 5 or more times per week*	1.44 (1.15, 1.80)	**0.001**	1.54
**Smoke cigarettes**	0.87 (0.72, 1.07)	0.187	0.87
**Drank within 3 months**			
* no*	–	–	–
* yes*	0.67 (0.55, 0.82)	**<0.001**	0.70
* unknown*	0.88 (0.74, 1.05)	0.148	0.91
**Military**			
* no*	–	–	–
* yes*	0.87 (0.70, 1.07)	0.192	0.94
* unknown*	0.83 (0.56, 1.24)	0.375	0.99
**Think about sex**			
* never*	–	–	–
* less than once a month*	0.64 (0.49, 0.83)	**<0.001**	0.76
* one to a few times a month*	0.66 (0.50, 0.88)	**0.004**	0.82
* one to a few times a week*	0.55 (0.41, 0.75)	**<0.001**	0.71
* everyday*	0.44 (0.31, 0.62)	**<0.001**	0.59
* other*	0.32 (0.20, 0.51)	**<0.001**	0.45
**Importance of sex**			
* not at all important*	–	–	–
* somewhat important*	1.35 (1.06, 1.72)	**0.017**	1.20
* moderately important*	1.60 (1.24, 2.07)	**<0.001**	1.36
* very important*	1.76 (1.33, 2.34)	**<0.001**	1.49
* extremely important*	2.31 (1.56, 3.41)	**<0.001**	1.82
* unknown*	1.52 (1.09, 2.11)	**0.014**	1.23
**Consensual sex**			
* sometimes*	–	**–**	–
* usually*	0.59 (0.42, 0.82)	**0.002**	0.76
* always*	0.60 (0.44, 0.83)	**0.002**	0.78
* partner hasn’t wanted sex/does not have partner*	0.74 (0.52, 1.06)	0.101	1.00
* unknown*	0.58 (0.41, 0.82)	**0.002**	0.79
* other*	0.85 (0.57, 1.26)	0.417	1.00
**Religion**			
* none*	–	–	–
* Catholic*	0.98 (0.72, 1.33)	0.883	Removed^2^
* other religion*	0.70 (0.51, 0.97)	**0.030**	0.75
* Protestant*	1.03 (0.77, 1.39)	0.837	1.05
* unknown*	1.28 (0.85, 1.93)	0.231	1.17
**Childhood: Family life was happy**			
* I disagree pretty much*	–	–	–
* I disagree a little*	0.94 (0.66, 1.35)	0.754	0.99
* I agree a little*	0.71 (0.49, 1.03)	0.074	0.75
* I agree pretty much*	0.72 (0.54, 0.98)	**0.037**	0.78
* I agree very much*	1.13 (0.81, 1.57)	0.483	1.20
* unknown*	1.31 (0.48, 3.72)	0.599	1.21
* other*	0.92 (0.58, 1.44)	0.703	0.98
**Childhood: Family was well off**			
* not well off at all*	–	–	–
* not so well off*	1.68 (1.30, 2.18)	**<0.001**	1.49
* about average*	1.48 (1.14, 1.91)	**0.003**	1.29
* fairly well off*	1.54 (1.11, 2.16)	**0.011**	1.36
* unknown*	1.79 (0.58, 5.21)	0.295	1.12
* other*	2.04 (1.09, 3.82)	**0.025**	1.82
**Childhood: Live with parents**			
* no*	–	–	–
* yes*	0.92 (0.73, 1.15)	0.453	0.94
* unknown*	0.66 (0.24, 1.80)	0.422	Removed^2^
**Childhood: Health**			
* good*	–	–	–
* very good*	0.90 (0.72, 1.13)	0.384	0.95
* excellent*	0.87 (0.70, 1.09)	0.219	0.95
* unknown*	6.12 (1.17, 38.1)	**0.038**	Removed^2^
* other*	1.47 (1.02, 2.13)	**0.039**	1.45
**Childhood: Experience violence**			
* no*	–	–	–
* yes*	0.52 (0.38, 0.72)	**<0.001**	0.54
* unknown*	0.29 (0.06, 1.17)	0.105	0.98
**Childhood: Witness violence**			
* no*	–	–	–
* yes*	0.88 (0.66, 1.18)	0.402	0.89
* unknown*	0.48 (0.11, 1.84)	0.291	Removed^2^
**Perceived Stress Scale 4** (PSS4)	1.06 (1.02, 1.10)	**0.003**	1.15
**NSHAP Depressive Symptoms Measure** (NDSM)	1.03 (1.01, 1.04)	**0.006**	1.11
**Functional difficulties**	1.04 (1.02, 1.06)	**<0.001**	1.39
**Hospital Anxiety and Depression Scale** (HADS)	1.03 (1.00, 1.06)	0.053	1.11
**Social disconnectedness** (SD)	1.50 (1.28, 1.77)	**<0.001**	1.23
**Perceived isolation** (PI)	0.92 (0.80, 1.06)	0.229	0.98

^1^OR = odds ratio; CI = confidence interval; categorical levels that are the reference group are denoted with –.

^2^Factor levels that were “removed” were combined with the reference level.

**Fig 4:**
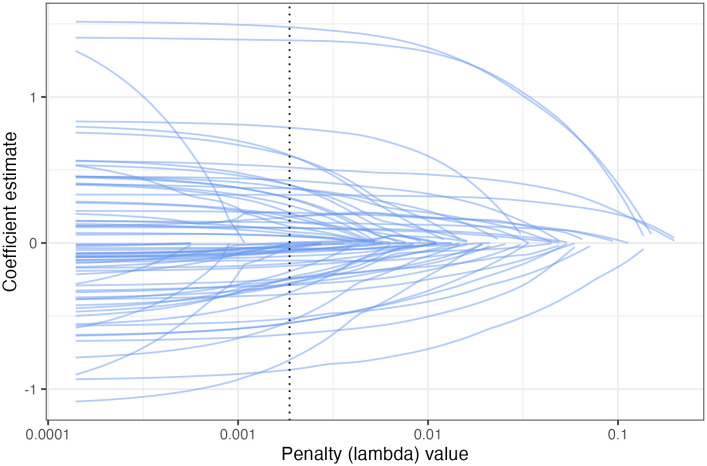
Coefficients of the elastic net model as penalization (lambda) increases.

The tuned elastic net model had a mixture of 0.623, meaning it was closer to a lasso regression model than to a ridge regression model. Fig 4 shows the 72 predictors being shrunk down to zero as the penalty (lambda) parameter increases. The dotted line represents the penalty parameter that had the best performance on the validation sets. With this penalty parameter, the 67 remaining variables are presented in Table 5 and the five variable which were removed are indicated with the “removed” label. The variable levels that were removed are the Catholic religion, unemployment due to disability, *unknown* if patient lived with parents in childhood, *unknown* if patient had good health in childhood, and *unknown* if patient witnessed violence in childhood. These unknown levels could be due to patients not responding or genuinely not remembering. Since these unknown levels did not contain useful information, they were likely missing at random rather than systematically missing. Recall that the three levels removed relating to childhood experiences were the variables identified by the logistic regression as having a high correlation. The elastic net model achieved the same performance as the logistic regression model while removing unnecessary information.

The MARS model (Equation 1) contained 18 of the 72 predictors and retained 35 of the 36 coefficients and hinge functions that it created. The coefficients for *functional difficulties* are large and numerous, indicating that the model has a strong reliance on that predictor. Despite functional difficulties’ large coefficient values, the model still produces reasonable predictions. For example, when there are no functional difficulties, then the term for functional difficulties offsets the large intercept (shown below), giving all other coefficients the ability to have an effect on the model.


0.036=6485.2−4244.5505*(1.52788−0)

When there is only one functional difficulty, the terms offset to the following.


$$\begin{array}{c} -\text{0}.\text{1}\text{0}\text{4}=\text{6}\text{4}\text{8}\text{5}.\text{2}\\ \;\;\;\;-\text{4}\text{2}\text{4}\text{4}.\text{5}\text{5}\text{0}\text{5}*(\text{1}.\text{5}\text{2}\text{7}\text{8}\text{8}-\text{1})\\ \;\;\;\;-\text{4}\text{3}\text{9}\text{4}.\text{2}\text{6}\text{1}\text{3}*(\text{1}-\text{0}.\text{1}\text{1}\text{2}\text{7}\text{4}\text{7})\\ \;\;\;\;-\text{1}\text{6}\text{2}\text{3}.\text{8}\text{2}\text{3}\text{2}*(\text{1}-\text{0}.\text{7}\text{8}\text{7}\text{0}\text{0}\text{3}) \end{array}$$

Equation 1. Multivariate adaptive regression splines model.


$$\begin{array}{cc} \text{ln}\left(\frac{p}{\text{1}-p}\right) & =\text{6}\text{4}\text{8}\text{5}.\text{2}\\ & \;-\text{0}.\text{5}\text{3}\times X_{\text{female}}\\ & \;+\text{1}.\text{5}\text{2}\times X_{\text{race}/\text{ethnicity}:\text{ Black}}+\text{1}.\text{5}\text{4}\times X_{\text{race}/\text{ethnicity}:\text{ Hispanic},\text{ non}-\text{black}}+\text{0}.\text{9}\text{0}\times X_{\text{race}/\text{ethnicity}:\text{ other}}\\ & \;-\text{0}.\text{3}\text{0}\times X_{\text{household income}:\text{ 25},\text{000}-\text{49},\text{999}}-\text{0}.\text{5}\text{7}\times X_{\text{household income}:\text{ 50},\text{000}-\text{99},\text{999}}-\text{0}.\text{9}\text{3}\times X_{\text{household income}:\text{ 100k or higher}}\\ & \;+\text{0}.\text{3}\text{1}\times X_{\text{widowed}}\\ & \;+\text{0}.\text{4}\text{0}\times X_{\text{physical activity}:\;\text{5}\text{ or more times per week}}\\ & \;-\text{0}.\text{3}\text{4}\times X_{\text{drank within }\text{3}\text{ months}}\\ & \;-\text{0}.\text{5}\text{6}\times X_{\text{family was happy growing up}:\text{ I agree a little}}-\text{0}.\text{4}\text{4}\times X_{\text{family was happy growing up}:\text{ I agree pretty much}}\\ & \;-\text{0}.\text{5}\text{3}\times X_{\text{experienced violence in childhood}}\\ & \;\left\{ \begin{array}{cc} X_{\text{age}}<\text{6}\text{4} & -\text{0}.\text{0}\text{4}\times \left(\text{6}\text{4}-X_{\text{age}}\right)\\ X_{\text{age}}>\text{6}\text{4} & +\text{0}.\text{0}\text{6}\times \left(X_{\text{age}}-\text{6}\text{4}\right)\\ X_{\text{age}}>\text{8}\text{8}.\text{5} & -\text{0}.\text{8}\text{6}\times \left(X_{\text{age}}-\text{8}\text{8}.\text{5}\right)\\ X_{\text{PSS4}}<\text{7}.\text{4}\text{5} & -\text{0}.\text{0}\text{4}\times \left(\text{7}.\text{4}\text{5}-X_{\text{PSS4}}\right)\\ X_{\text{PSS4}}>\text{7}.\text{4}\text{5} & +\text{0}.\text{0}\text{6}\times \left(X_{\text{PSS4}}-\text{7}.\text{4}\text{5}\right)\\ X_{\text{functional difficulties}}>\text{0}.\text{1}\text{1} & -\text{4}\text{3}\text{9}\text{4}.\text{3}\times \left(X_{\text{functional difficulties}}-\text{0}.\text{1}\text{1}\right)\\ X_{\text{functional difficulties}}>\text{0}.\text{7}\text{9} & -\text{1}\text{6}\text{2}\text{3}.\text{8}\times \left(X_{\text{functional difficulties}}-\text{0}.\text{7}\text{9}\right)\\ X_{\text{functional difficulties}}>\text{1} & \text{1}\text{8}\text{0}\text{4}.\text{2}\times \left(X_{\text{functional difficulties}}-\text{1}\right)\\ X_{\text{functional difficulties}}<\text{1}.\text{5}\text{3} & -\text{4}\text{2}\text{4}\text{4}.\text{6}\times \left(\text{1}.\text{5}\text{3}-X_{\text{functional difficulties}}\right)\\ X_{\text{functional difficulties}}>\text{1}.\text{5}\text{3} & \text{4}\text{1}\text{7}\text{9}.\text{9}\times \left(X_{\text{functional difficulties}}-\text{1}.\text{5}\text{3}\right)\\ X_{\text{functional difficulties}}>\text{2} & \text{1}\text{1}\text{6}.\text{3}\times \left(X_{\text{functional difficulties}}-\text{2}\right)\\ X_{\text{functional difficulties}}>\text{2}.\text{1}\text{4} & -\text{9}\text{5}.\text{9}\times \left(X_{\text{functional difficulties}}-\text{2}.\text{1}\text{4}\right)\\ X_{\text{functional difficulties}}>\text{3} & \text{5}\text{0}.\text{1}\times \left(X_{\text{functional difficulties}}-\text{3}\right)\\ X_{\text{functional difficulties}}>\text{3}.\text{0}\text{9} & -\text{3}\text{4}.\text{6}\times \left(X_{\text{functional difficulties}}-\text{3}.\text{0}\text{9}\right)\\ X_{\text{functional difficulties}}>\text{3}.\text{5}\text{4} & -\text{1}\text{1}.\text{4}\times \left(X_{\text{functional difficulties}}-\text{3}.\text{5}\text{4}\right)\\ X_{\text{functional difficulties}}>\text{4} & \text{4}\text{1}.\text{1}\times \left(X_{\text{functional difficulties}}-\text{4}\right)\\ X_{\text{functional difficulties}}>\text{4}.\text{1}\text{1} & -\text{3}\text{5}.\text{2}\times \left(X_{\text{functional difficulties}}-\text{4}.\text{1}\text{1}\right)\\ X_{\text{functional difficulties}}>\text{5} & \text{3}.\text{4}\text{2}\times \left(X_{\text{functional difficulties}}-\text{5}\right)\\ X_{\text{HADS}}<\text{9}.\text{5}\text{4} & -\text{0}.\text{0}\text{6}\times \left(\text{9}.\text{5}\text{4}-X_{\text{HADS}}\right)\\ X_{\text{SD}}<\text{0}.\text{5}\text{7} & -\text{0}.\text{6}\text{1}\times \left(\text{0}.\text{5}\text{7}-X_{\text{SD}}\right)\\ X_{\text{SD}}>\text{0}.\text{5}\text{7} & -\text{0}.\text{7}\text{1}\times \left(X_{\text{SD}}-\text{0}.\text{5}\text{7}\right) \end{array}\right.\; \end{array}$$

**Fig 5:**
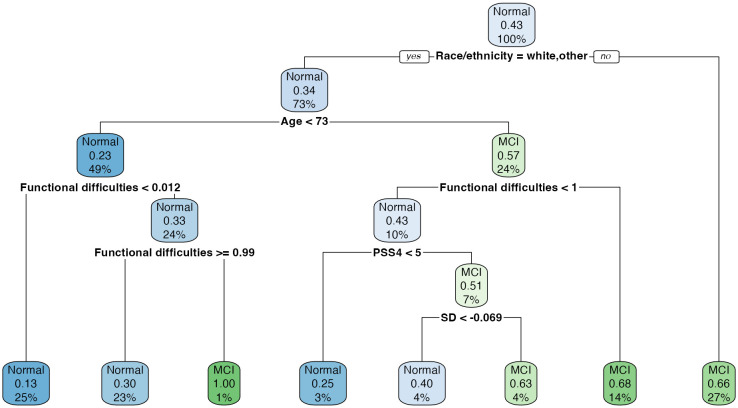
Decision tree model.

**Fig 6:**
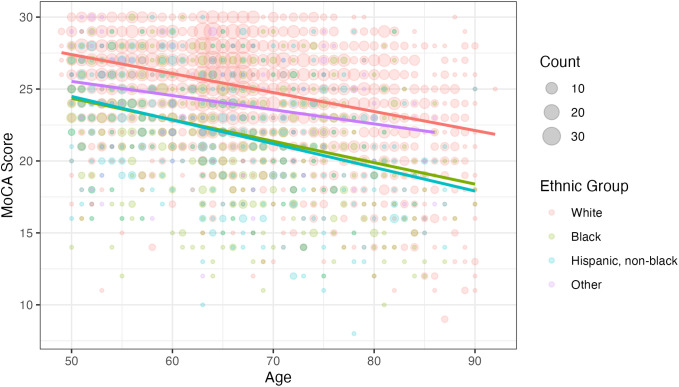
MoCA scores by age and ethnicity.

The decision tree shown in Fig 5 demonstrates that most of the variation in MCI classification can be explained by age, ethnicity, and functional difficulties. The leftmost branch of the diagram contains some redundancy that could be simplified. Specifically, individuals who are White or other ethnicity and younger than 73 can be classified into two categories – MCI if they have one or more functional difficulties, or normal if they have no functional difficulties. On the other side of the decision tree, stress and SD play a role for those who are White or other ethnicity, 73 years or older, and have no functional difficulties. Fig 6 explores how the first two variables of the decision tree relate to MoCA scores using the complete dataset. Points and linear regression lines are plotted for each ethnicity. Age shows an inverse relationship with MoCA scores, with Whites generally scoring higher than Blacks or Hispanics. The *other* ethnicity does not experience as steep a decline in MoCA scores as age increases, compared to the other ethnicities.

**Fig 7:**
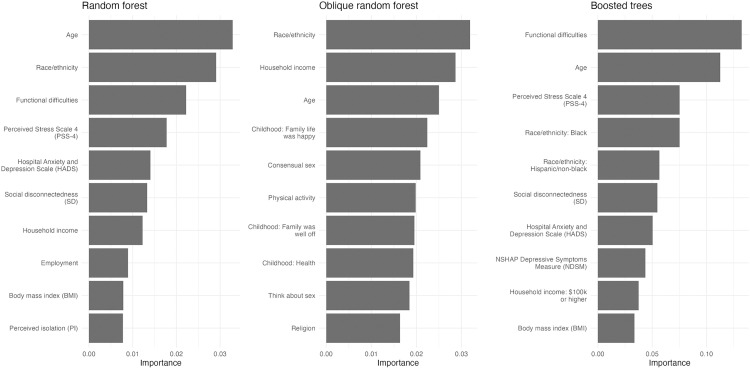
Variable importance plots of the random forest, oblique random forest, and boosted trees models.

Random forest, oblique random forest, and boosted trees are extensions of the decision tree model. The variables that are most important to each model are shown in Fig 7. The most important variables of the random forest model are similar to those in the MARS and decision tree model. An emphasis is placed on age, race/ethnicity, functional difficulties, stress, and SD. The oblique random forest used many childhood factors such as if the patient was healthy or if their family was happy or well off. The boosted trees model used many lifestyle-related scales measuring stress, SD, anxiety, and depression. All three models listed BMI as a useful feature while the logistic and elastic net models did not have significant coefficients for BMI.

**Fig 8:**
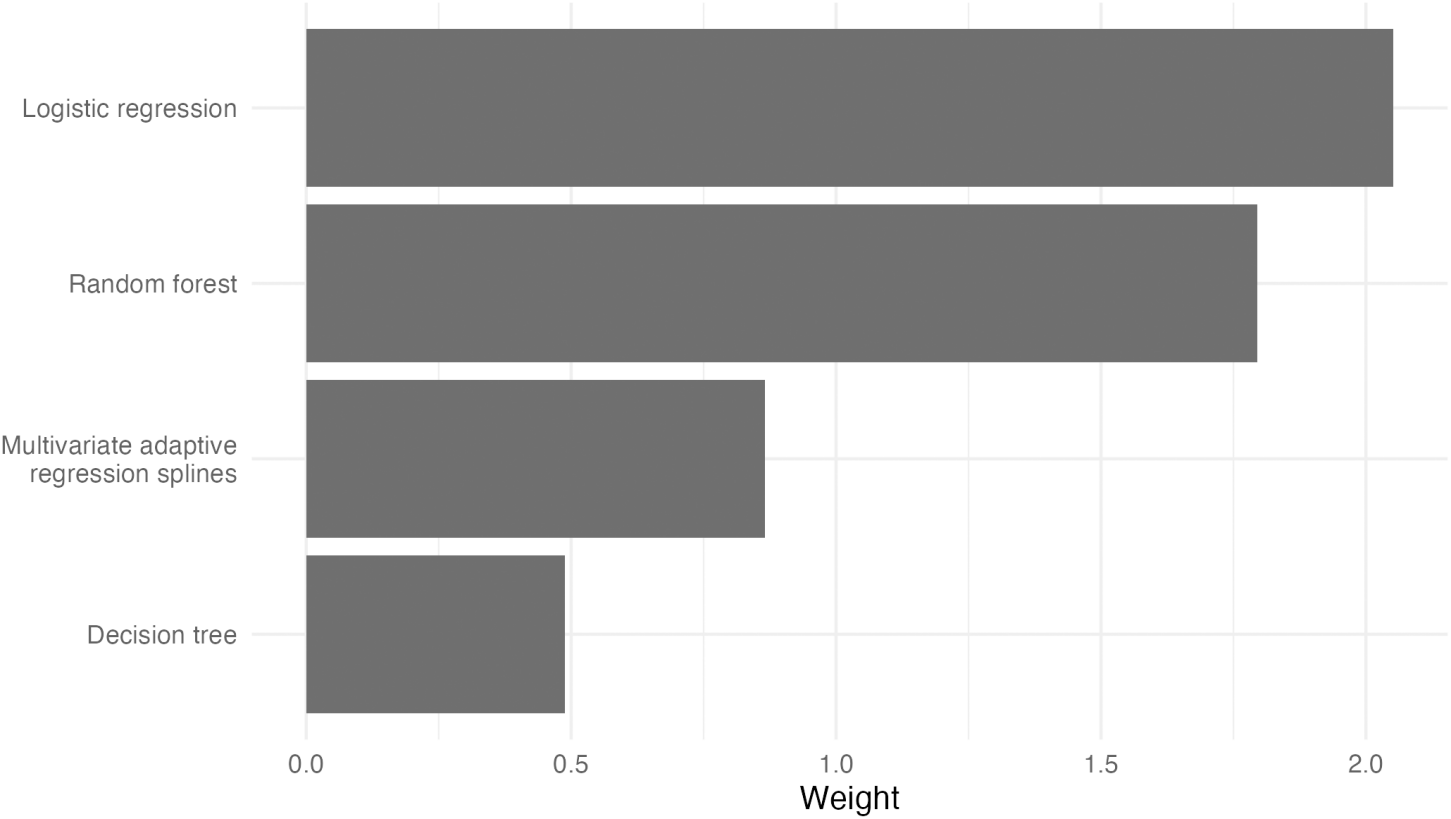
Model weights for the stacked ensemble model.

Four models were retained by the lasso regression model of the stacked ensemble (Fig 8). This ensemble achieved the best AUROC and MCC compared to all other models. Logistic regression (weight = 2.05) exhibited the best standalone performance and was given the highest weight by the lasso model. Interestingly, random forest (weight = 1.79) was selected over more complex tree-based models, such as oblique random forest and boosted trees, despite their better performance. This may be because random forest offered greater regularization due to its simplicity. Models that provide better interpretability and more granular predictions, such as MARS (weight = 0.87) and decision tree (weight = 0.49), were also selected.

## Discussion

Nearly all machine learning models agreed that age, ethnicity, functional difficulties, SD, and stress were the most important variables, as determined by their variable importance or inclusion in each model. These findings corroborate with previous research. Age is recognized as the greatest risk factor for dementia [27] and difficulty with activities of daily living is part of the diagnostic criteria for dementia [28]. Lack of social support is a known risk factor for dementia [2], and a study using the NSHAP found that SD was associated with a higher prevalence of MCI [29].

Dementia prevalence and incidence vary among ethnic groups. Research shows that dementia incidence and prevalence are higher in minority ethnic groups when assessed through population-based surveys rather than electronic health records, highlighting the importance of using culturally appropriate cognitive screening tools [30]. Machine learning models using populational data could potentially help to identify patients who are at high risk for the development of mild cognitive impairment and dementia in a manner that is relatively free of cultural bias; however, machine learning could also perpetuate societal biases in data, so this would need to be explored with caution.

The relationship between stress and cognitive impairment is not as well-explored as the other variables. Some research suggests that stress may be a risk factor for dementia [31]. Animal models suggest that stress can trigger increases in amyloid beta plaque and tau tangles that are the pathological hallmarks of Alzheimer’s disease perhaps through increases in cortisol [32]. Additional research will be beneficial in further elucidating possible associations and their mechanisms.

Ethnicity presented itself with the largest ORs in the logistic regression model. Regularization models such as elastic net, MARS, and decision trees utilized age and ethnicity while complex tree-based models such as random forest, oblique forest, and boosted trees listed age and ethnicity within their top four important variables. Similarly, every model from the ML study by Li, Zeng (7) chose age and ethnicity as their first and second most important variables, respectively. In a Singaporean ML study of multi-ethnic Asians, age, followed by education and then ethnicity were the most important predictors of cognitive decline. In the Northern Manhattan Study, a clinical evaluation by neuropsychologists and neurologists determined that Blacks and Hispanics had a 20% greater likelihood of having MCI compared to Whites, adjusting for age and education [33]. Blacks in this study had a similar disposition to MCI. Differences in education by race could have been a driving factor, but education could not be included as a predictor because it was used to adjust MoCA scores. Another study found that blood-based and physiological biomarkers only accounted for 22.7–35.2% of the ethnic differences in cognition [34], so further work can be done to understand the underlying pathways which put Blacks and Hispanics at extreme odds for cognitive impairment.

The decision tree created from the *rpart* package [35] is an implementation of Breiman’s method [36]. To create a split, or *node*, in the decision tree represents the variable and cut point that minimizes the misclassification error. The data is continually split until the reduction in misclassification error of the node is less than that of the upstream node [37]. To prevent under- or over-fitting, cross-validation is used to prune the tree by eliminating poorly performing nodes based on a cost-complexity parameter. The most important variables based on this study’s decision tree were age, ethnicity, functional difficulties, stress, and SD. These variables were either selected or considered very important by all models in this study.

Random forest improves on the decision trees model by adding regularization to the predictors. This is done by creating many trees in which the nodes of each tree select a random subset of the predictors from the data. In the end, an ensemble of trees is created in which the majority vote determines the ensembles prediction. Oblique random forest improves on random forest by creating a linear combination of the predictors selected at each node, thereby capturing more complex interactions. Lastly, boosted trees starts with a standard random tree, but trains the next tree on the residuals of the current tree. Each tree sequentially lowers the error of the overall model by correcting the error of the previous tree. Ultimately, none of these tree-based models performed as well as logistic regression.

Many models also had a reliance on functional difficulties. One study found that *physical frailty* was associated with MCI and quicker cognitive decline [38]. In their study, Boyle et al based physical frailty on grip strength, timed walk, body composition, and fatigue and monitored these characteristics over the course of 12 years annually. Interestingly, oblique random forest – which uses linear combinations of two predictors at a time – does not list functional difficulties as one of its top ten important variables. The permutation method of variable importance may not have captured the complex relationships between all predictors due to only permutating one variable at a time.

The MARS model had a particularly strong reliance on functional difficulties – an occurrence that could be explained by the model’s greedy algorithm which iteratively creates hinge functions for the predictor that reduces the most loss. It appears that the model’s *backward* pruning method did not remove many of functional difficulties hinge functions. A sensitivity analysis was conducted without the functional difficulties predictor to observe any difference in model performance or equation. The model achieved an AUROC of 0.806 (0.774, 0.836) which is slightly lower than its original performance, 0.815 (0.785, 0.844). The model’s reliance on functional difficulties was replaced with the NDSM (depression scale) which was not in the original model. This variable had extreme coefficients across three hinge functions which is a decrease from the original model’s 13 hinge functions. Conversely, when the sensitivity analysis was applied to the logistic model, the model’s performance was nearly the same with an AUROC of 0.817 (0.787, 0.846) and coefficients that were similar in terms of magnitude and significance.

All models considered SD as important, either by its p-value, its inclusion, or its variable importance. However, PI was not significant in logistic regression and was not included in the decision tree model. In contrast, a systematic analysis conducted by Cardona and Andres [39] found that both SD and PI were associated with cognitive decline, with depression being a possible mediator. Perhaps PI’s insignificance could be explained by the inclusion of other introspective measurements such as anxiety, depression, and stress which proved to be stronger predictors. Anecdotally, a study of 31 elderly persons with MCI and their 13 caregivers noted that their immobility, lack of activities, and feelings of close-minded communities had affected their ability to create social connections [40]. This relationship may explain why functional difficulties and SD play a strong role in this study.

Although the ensemble achieved the highest point estimate of performance, its use in clinical practice is contingent on the scenario. For example, in time-critical situations such as detecting cardiac arrest [41] or sepsis [42] during hospitalization, detecting events sooner can initiate treatment that will improve patient outcomes. Medical practices already have a standard protocol for diagnosing patients. In these situations, quickly alerting clinicians to confirm their *own* diagnosis takes precedence over immediate interpretability of the algorithm. However, fields such as research will always prioritize explainability over marginal performance gains because that is the nature of understanding how features are related. The goal of this study is to detect the start of mental decline and allow clinicians to screen earlier for it, so clinicians also need to know what to look for. For that reason, the logistic regression model provides the best balance of interpretability and performance that clinicians find practical.

Our results should be interpreted with the following limitations. Low scores on the MoCA were used as a proxy for MCI, but because the MoCA is only a screening tool, it should not be used alone to diagnose MCI and dementia and should be accompanied by a clinical evaluation. Ideally, cognitive decline is monitored relative to their baseline cognitive performance and everyday lifestyle [43]. This study is cross sectional because there were not enough study participants who completed the MoCA in Wave 2 and 3 of the NSHAP, and, unfortunately, the NSHAP data do not include clinically confirmed diagnoses of MCI or dementia. The severity and domain(s) of cognitive impairment are not determined in this study. Additionally, education could not be assessed as a risk factor because MoCA scores were adjusted based on the highest level of education achieved. Data from the NSHAP may not be generalizable to other countries; the prevalence of risk factors for cognitive impairment and the amount of risk that these factors confer vary between countries, ethnic groups, and socioeconomic statuses [30]. Next, the permutation method used to determine variable importance may not have been appropriate for the oblique random forest model because each node was a combination of predictors. However, this method was chosen so that all three tree-based models could have a fair comparison of variable importance. Lastly, complex survey design – weights, strata, and cluster – were not incorporated into the models. The NSHAP purposefully oversampled minorities such as Blacks and Latinos to improve population estimations [44]. To account for this, their weights are lower than other majorities. Since this study did not incorporate weights, the results of the models are biased toward minority populations and not completely generalizable to the entire US older population. A sensitivity analysis was conducted, incorporating weights into logistic regression’s modeling, calibration, and final performance. The performance was nearly the same – an AUROC of 0.812 (0.784, 0.842) compared to the original 0.818 (0.788, 0.847).

Future research can be done with more complex data and machine learning models such as neural nets. The task of choosing a suitable architecture for the neural net and tuning it deserves a separate project of its own. Although this project uses many predictors, it would not be classified as a problem of “high dimensionality” where the number of predictors greatly exceeds the number of observations. Future studies would benefit from using longitudinal data to track cognitive decline with respect to each person’s baseline MoCA performance. Since functional difficulties was a strong predictor in this study, longitudinal observation could reveal the general direction of its relationship with cognitive impairment. For greater generalizability, survey weights could be incorporated into the models and performance calculations. Advanced variable importance techniques such as Shapley values could be used to interpret models which is crucial if any complex model outperforms traditional logistic regression.

## Conclusion

This study demonstrates that machine learning models can effectively use personal, social, and functional lifestyle factors to predict MCI, with performance comparable to traditional logistic regression. Across models, age, ethnicity, functional difficulties, social disconnectedness, and stress consistently emerged as key predictors, underscoring their central role in cognitive health. While the stacked ensemble model achieved the highest overall performance, the relatively strong showing of simpler models like logistic regression highlights that interpretability and accuracy can coexist, offering practical value for clinical applications.

Importantly, these findings affirm prior research linking social and lifestyle factors to cognitive outcomes, while also raising new considerations. For example, the predictive power of stress and SD suggests that psychosocial factors should be considered when screening MCI. However, the cross-sectional nature of this analysis, the reliance on screening rather than clinical diagnosis, and the absence of educational factors due to MoCA scoring adjustments limit the broader generalizability of these results.

Future research should leverage longitudinal datasets, include clinically confirmed diagnoses, and explore more advanced modeling techniques such as neural networks to refine risk prediction. Ultimately, integrating machine learning with culturally appropriate screening tools may help detect adults at risk for cognitive decline—providing an opportunity for timely intervention and support.
